# Exploring the Functional Role of Programmed Death‐Ligand 1 (PD‐L1) in the Castration‐Resistant Prostate Cancer Using Transcriptomic Sequencing Analysis

**DOI:** 10.1002/cam4.71225

**Published:** 2025-09-09

**Authors:** Lin Zhong, Pengxin Zhang, Jialin Ji, Jun Mao, Lianhong Li

**Affiliations:** ^1^ Department of Pathology First Affiliated Hospital Dalian Medical University Dalian China; ^2^ The Key Laboratory of Tumor Stem Cell Research of Liaoning Province Dalian Medical University Dalian China; ^3^ Department of Pathology and Forensics Dalian Medical University Dalian China

**Keywords:** altinative splicing, CRPC, PD‐L1, prostate cancer, transcriptomic sequencing

## Abstract

**Background:**

Prostate cancer is one of the principal malignancies threatening human health, and the development of castration resistance often constitutes a major cause of treatment failure in its management.

**Methods:**

To elucidate the potential association between programmed death‐ligand 1 (PD‐L1) and castration resistance in prostate cancer, we analyzed the expression levels of PD‐L1 in both primary prostate cancer tissues and castration‐resistant prostate cancer (CRPC) specimens as well as in corresponding cell lines by using western blots and immunohistochemistry. Then, we explored the specific mechanisms through transcriptomic sequencing technology.

**Results:**

Our findings revealed that, compared to adjacent non‐cancerous tissue, PD‐L1 expression was unexpectedly lower in primary prostate cancer but notably elevated in CRPC tissues and cells. In CRPC cell lines where PD‐L1 was knocked down, a significant suppression of proliferation, invasion, and migration capabilities was observed. By employing next‐generation sequencing technology, we investigated the impact of PD‐L1 knockdown on intracellular signaling pathways in castration‐resistant cells. The results demonstrated that PD‐L1 knockdown led to alterations in gene expression within several signaling pathways, including those involved in cell surface interactions, regulation of natural killer cell activity, and sodium channel regulatory activity. We further elucidated through experimentation that PD‐L1 contributes to tumor progression in CRPC by modulating the expression of SCUBE1. More intriguingly, PD‐L1 knockdown also appeared to induce changes at the level of alternative splicing in multiple genes.

**Conclusions:**

PD‐L1 is upregulated in CRPC and can modulate the expression of multiple tumor‐associated genes in CRPC cells. Finally, we found that PD‐L1 contributes to tumor progression in CRPC by modulating the expression of SCUBE1. This study provides a theoretical basis for understanding the intracellular signaling mediated by PD‐L1 and offers valuable insights into the mechanisms underlying castration resistance in prostate cancer.

## 1 Introduction

Prostate cancer ranks among the most prevalent cancers worldwide, with a significant number of fatalities attributed to the disease annually [1, 2]. Age, race, family history of the disease, and genetic mutations are recognized as direct risk factors associated with the development of prostate cancer; meanwhile, unhealthy lifestyle habits such as smoking and obesity are also implicated in increasing the likelihood of developing the condition [3]. Current treatments for prostate cancer mainly involve surgical resection, androgen deprivation therapy, as well as radiation and chemotherapy. Despite generally favorable overall prognoses, recurrence and metastasis of the tumor remain key contributors to mortality. Hence, identifying the causes and pathogenic mechanisms underlying recurrence and metastasis, as well as discovering novel therapeutic targets and predictive biomarkers, is of critical importance in improving patient outcomes [4, 5].

Androgen Deprivation Therapy (ADT) for prostate cancer, also known as androgen deprivation or castration therapy, can be categorized into two main approaches: surgical castration (bilateral orchiectomy) and medical castration (using Gonadotropin‐Releasing Hormone agonists or antagonists) [6]. This form of treatment serves as a neoadjuvant or adjuvant therapy option for certain locally advanced disease stages and constitutes a principal mode of treatment for patients with metastatic prostate cancer. ADT typically achieves a profound response, with around 90% of patients experiencing a decrease in their Prostate‐Specific Antigen (PSA) levels to normal or undetectable levels, and over 80% demonstrating objective tumor regression. However, despite its initial effectiveness, long‐term outcomes are often compromised due to the emergence of castration‐resistant prostate cancer (CRPC), where prostate cancer cells adapt and continue to grow despite low or absent levels of circulating androgens. This resistance represents a significant challenge in the management of advanced prostate cancer, necessitating the development of new therapeutic strategies to combat disease progression in this stage.

Castration‐resistant prostate cancer (CRPC): This refers to prostate cancer that continues to progress despite ongoing androgen deprivation therapy. The main underlying causes of its development include the following points: (1) Despite castration treatment, intratumoral steroidogenesis contributes to the synthesis of testosterone and dihydrotestosterone (DHT), potentially leading to persistent androgen levels within the tumor tissue [7]. (2) Mutations or amplification in the number of copies of the androgen receptor (AR) can result in enhanced affinity for DHT and dihydrotestosterone, thereby engendering resistance to castration‐based therapies [8, 9]. (3) Non‐ligand activation of AR can occur through non‐ligand‐dependent modifications or crosstalk with other signaling pathways, such as phosphorylation‐induced hypersensitization of AR and increased nuclear translocation, as well as cross‐talk with epidermal growth factor receptor (EGFR) [10]. (4) Altered balance between coactivators and corepressors of AR leads to an increase in AR activity. (5) Upregulation of anti‐apoptotic molecules, such as Bcl‐2, allows bypassing of the AR‐receptor‐dependent pathway, enabling tumor cell survival and the development of resistance [11].

PD‐L1 (B7‐H1, CD274) is a cell surface glycoprotein belonging to the B7 family that serves as a ligand for PD‐1, expressed on the surfaces of immune cells and various types of tumor cells [12]. The interaction between PD‐1 and PD‐L1 suppresses the functionality of cytotoxic T lymphocytes, playing a pivotal role in dampening immune responses. In recent years, research has revealed that the expression of PD‐L1 is regulated by signaling pathways, transcription factors, and epigenetic modifiers; this protein not only enables cancer cells to evade immune surveillance but also promotes their proliferation. PD‐L1 expression constitutes a critical biomarker predictive of disease progression and prognosis across multiple cancer types [13].

PD‐L1 can be expressed in prostate cancer, especially within the context of castration‐resistant prostate cancer (CRPC) tissue, with its clinical and biological significance still being elucidated. Christian and colleagues' research demonstrates that PD‐L1 expression is limited in castration‐resistant prostate cancer (CRPC), with no expression detected in localized prostate cancer (PC) or benign prostatic hyperplasia [14]. However, contrasting findings were reported by Francesco Massari et al., who showed that approximately 19% of CRPC patients exhibit PD‐L1 expression [15]. Further, Gevensleben and associates also found high expression levels of PD‐L1 in prostate cancer [16]. These conflicting results underscore the complexity of PD‐L1 expression patterns in prostate cancer and highlight the need for further investigation to better understand its implications in the progression to castration resistance and the potential utility of PD‐L1‐targeted therapies in this subset of patients.

Phosphatase and tensin homolog (PTEN) located on chromosome 10, is a dual lipid and protein phosphatase that dephosphorylates PIP3. By doing so, PTEN negatively regulates the PI3K/Akt pathway through deactivation of PIP3, thereby inhibiting PI3K activity and leading to downregulation of Akt [17, 18]. The PI3K/Akt pathway is a crucial signal transduction route promoting cell growth and survival, which is implicated in evasion of apoptosis, loss of cell cycle control, and genomic instability during tumorigenesis. It also impacts cellular differentiation, actin cytoskeleton rearrangement, and membrane trafficking [19, 20]. Multiple studies have demonstrated that PTEN loss in triple‐negative breast cancer and colorectal cancer can lead to upregulation of PD‐L1 expression at either the transcriptional or post‐transcriptional level through the PI3K/Akt signaling pathway [21, 22]. Other studies show that PTEN loss (along with TP53 and RB1 loss) is associated with poor prognosis in prostate cancer and is involved with lineage plasticity and transdifferentiation to neuroendocrine prostate cancer [23, 24, 25]. Overexpression of PD‐L1 in these tumors has been associated with increased risks of progression and metastasis, as well as stemness properties of the cancer cells, contributing to drug resistance. Regarding castration‐resistant prostate cancer (CRPC), however, there is a lack of reported evidence on the specific role that PD‐L1 overexpression plays in mediating resistance mechanisms in CRPC, as well as its influence on the stemness properties of CRPC cells. Research in this area could provide insights into potential therapeutic strategies targeting the PD‐L1 axis in the context of CRPC and help elucidate how the interplay between PTEN, PI3K/Akt signaling, and PD‐L1 expression influences the aggressive behavior and treatment resistance of CRPC.

Recent research has unveiled that PD‐L1 can exert multifaceted effects on cancer cells expressing it, influencing their growth, survival, stem cell‐like properties, DNA damage response, and gene regulation [26]. Historically, much focus has been placed on the extracellular signaling mediated by PD‐L1 while its intracellular signaling functions have received less attention. The PD‐L1 protein comprises five major protein domains, and when referring to the full‐length PD‐L1 protein in humans, it is noted that not only does it mediate intracellular signaling when expressed on the cell surface, but it also modulates a variety of signaling pathways within the cell itself [27, 28, 29]. For instance, a discovery in 2008 highlighted the critical role played by the cytoplasmic tail of PD‐L1 in its intracellular anti‐apoptotic function [30]. Given these findings, to investigate whether PD‐L1 indeed exerts its functions via intracellular signaling that affects the survival and drug resistance of tumor cells, the following research was conducted.

Based on the aforementioned background, we investigated the correlation between PD‐L1 and CRPC, as well as its intracellular functions. Our results hold potential clinical relevance and value, providing insights into possible therapeutic interventions targeting PD‐L1 and its downstream effects in CRPC.

## 2 Materials and Methods

### 2.1 Cell Culture

Cell lines used in this study were obtained from the American Type Culture Collection (ATCC) and cultured under standard culture conditions in culture medium recommended by the ATCC. PC3 cells were maintained in F12K (cat. no. SH30526.01; Cytiva) with 10% FBS (cat. no. FBS‐Superior‐L; Sagecreation Co.). DU145 cells were cultured with EMEM (cat. no. PWL104; meiluncell) media supplemented with 10% FBS, while LNCAP cells were maintained using 1640 (cat. no. 6124150; Gibco) media supplemented with 10% FBS.

To achieve a stable knockdown of PD‐L1 in post‐castrate resistant prostate cancer (post‐CRPC) cells, lentiviral vectors were employed. Following the manufacturer's instructions, we co‐transfected 293T cells with the pLKO.1‐PD‐L1‐sh construct (using an empty pLKO.1 vector as a control) alongside PAX2 and PMD2. The viral supernatant was harvested after removing cellular debris via centrifugation. Post‐CRPC cells were then infected with these viral particles, and stable integration was achieved by selecting the cells with 2 μg/mL puromycin over a period of 5 days. Subsequently, the cells were cultured in medium supplemented with 1 μg/mL puromycin at 37°C under 5% CO_2_ in a humidified incubator. Before proceeding with further analyses, all stable cell lines were verified using Western blotting. The primers used for constructing the knockdown plasmids are listed below:

PD‐L1‐SH‐F: CCGGCGAATTACTGTGAAAGTCAATCTCGAGATTGACTTTCACAGTAATTCGTTTTTG

PD‐L1‐SH‐R: AATTCAAAAACGAATTACTGTGAAAGTCAATCTCGAGATTGACTTTCACAGTAATTCG

To establish a PC3 cell line with both PD‐L1 knockdown and SCUBE1 overexpression, we first constructed a PD‐L1‐knockdown PC3 cell line using the aforementioned method. Subsequently, on the basis of the PD‐L1‐knockdown PC3 cells, we further introduced a virus carrying SCUBE1 to achieve overexpression of SCUBE1, thereby generating a PC3 cell line with concurrent PD‐L1 knockdown and SCUBE1 overexpression.

### 2.2 Transwell Assay

The assessment of cell migration was conducted utilizing a transwell assay. The experiment involved chambers placed within a 24‐well culture plate (cat. no. CLS3422; Corning), with 600 μL of complete medium supplemented with 20% FBS situated in the lower compartment. Subsequently, 5 × 10^4^ prostate cancer cells were diluted in 100 μL of a medium devoid of serum and introduced into chambers featuring an 8 μm polycarbonate membrane, with each condition tested in three replicates. Incubation proceeded at 37°C in an atmosphere containing 5% CO_2_ for 24 h. Following this, the chambers were fixed using 4% paraformaldehyde and stained with a 0.5% crystal violet solution for 15 min. Cells that did not migrate remained on the interior side of the membrane and were removed using a cotton swab. Migrated cells adhering to the underside of the membrane were visualized using a standard bright‐field microscope outfitted with a digital camera.

### 2.3 Scratch Wound Healing Assay

The scratch wound healing assay assesses cell migration by creating a gap in a confluent cell monolayer using a sterile pipette tip. After washing away debris with PBS, cells are incubated in fresh medium, typically at 2% serum levels. Images of the scratch are captured at various time points (0, 24, 36 h) using a microscope equipped with a digital camera. The rate of wound closure is then quantified to evaluate cell migration efficiency.

### 2.4 Western Blot

Cells were washed with cold PBS and then resuspended in RIPA lysis buffer (cat. no. R0020; Solarbio). The cell lysates were centrifuged at 12000 rpm for 20 min, and the protein concentration was measured using a BCA protein assay kit (cat. no. P0011‐1; Beyotime). Equal amounts of total protein were mixed with loading buffer and denatured for 5 min at 95°C. Proteins (30 μg) were loaded onto SDS‐polyacrylamide gels (10%) and transferred to a nitrocellulose membrane. The membranes were blocked with 4% fat‐free milk and incubated with primary antibodies at 4°C overnight. After PBS washes for 3 times, the membranes were incubated with a secondary antibody (cat. no. 111‐035‐003; jackson immunoresearch) for 1 h at room temperature. Protein bands were visualized with the ECL kit (cat. no. Q02003; Sagecreation) and finally, MINICHEMI (MiniChemi 580; Sinsagetech Co. Ltd.) was used for visualization. Vinculin was used as the loading control. Image J software was used to analyze the grayscale values of western bands. The following antibodies were used: PD‐L1 (CST, 13684s) and vinculin (proteintech, 66305‐1‐Ig).

### 2.5 Patients and Tissue Sample

Patients were selected from those enrolled in the department of pathology, first affiliated hospital Dalian Medical University from 2017 to 2024. Subjects were selected by the following criteria: (1) The ADPC group mostly included patients initially diagnosed with prostate cancer, untreated. (2) The CRPC group comprised patients with persistent elevation of serum total PSA (tPSA) levels after normalization during androgen deprivation therapy, or imaging evidence supporting tumor progression. In total, 68 ADPC and 10 CRPC patients were enrolled. The TNM stage was reviewed according to the TNM staging system of the World Health Organization (WHO) standard. Clinicopathologic data were shown in Table 1. The study was approved by the Ethical Committees of the first affiliated hospital Dalian Medical University (PJ‐KS‐KY‐2002‐452).

**TABLE 1:** Clinicopathologic data of patients.

Group pathological feature	CRPC	ADPC
Age (average)	72.0 ± 7.8	67.7 ± 10.0
tPSA (average)	169.2 ± 401.1	128.7 ± 147.3
Metastases (%)	38.7	88.9
High Gleason score (%)	40.3	0
Middle Gleason score (%)	31.3	30
Low Gleason score (%)	28.4	70
Acinar adenocarcinoma (%)	98.5	80
Ductal adenocarcinoma (%)	1.5	20
Smoking history (%)	29	28.6
TNM (T1 + 2) (%)	69.8	50
TNM (T3 + 4) (%)	30.2	50
Diabetes history (%)	21.9	14.3
Hypertension history (%)	48.4	14.3
Coronary heart disease history (%)	10.9	0
Alcohol history (%)	11.3	14.3

Inclusion criteria (ADPC):

1. Serum testosterone levels reached castrate levels (< 1.7 nmol/L)
2. The tumor demonstrated a response to treatment.

Inclusion criteria (CRPC):

1. Serum testosterone levels reached castrate levels (< 1.7 nmol/L)
2. PSA (prostate specific antigen) levels were measured three consecutive times with intervals of 1 week or more, showing an increase of more than 50% above the lowest recorded value, with PSA > 2 μg/L.

Exclusion criteria:

1. The patient was diagnosed with additional infectious conditions, including prostatitis, which necessitate consideration in the overall management plan.
2. The patient did not sign the informed consent form.

### 2.6 Immunohistochemical

Enhance labeled polymer system (ELPS) was used for immunohistochemistry. 4 μm sections were prepared from paraffin‐embedded tissue blocks, deparaffinized, and rehydrated. Antigen retrieval was performed using a citrate buffer solution and heat, followed by blocking of endogenous peroxidase activity and non‐specific binding sites. The sections were incubated with the primary antibody overnight at 4°C, and then incubated with a secondary antibody conjugated with the ELPS label for 30 min at room temperature. The signal was enhanced with the polymer enhancer reagent, and the color was developed using DAB substrate, followed by counterstaining with hematoxylin. Finally, the sections were dehydrated and mounted with coverslips using mounting medium. PD‐L1 (AmoyDx cat# E1L3N) PTEN (MAXIM cat# 138G6) CD133 (ABCAM cat# AB19898) CD44 (ZSBIO cat# OTI1D8) were used.

### 2.7 RNA‐Seq

RNA was extracted from DU145 and DU145 PD‐L1sh1/2 cells using TRIzol Reagent (Ambion). Subsequently, RNA‐Seq analysis was carried out by Novogene. First, total RNA samples are quality checked. After ensuring the samples meet the criteria, mRNA is enriched from eukaryotic cells using magnetic beads with Oligo(dT). Subsequently, fragmentation buffer is added to break down the mRNA into short fragments. Using these mRNA fragments as templates, single‐stranded cDNA (ss‐cDNA) is synthesized with random hexamer primers. Then, buffer, dNTPs, and DNA Polymerase I are added to synthesize double‐stranded cDNA (ds‐cDNA). The ds‐cDNA is purified using AMPure XP beads. The purified ds‐cDNA undergoes end repair, A‐tailing, and adapter ligation. Size selection of the fragments is performed using AMPure XP beads, followed by PCR amplification to enrich the final cDNA library. After library construction, initial quantification is performed using Qubit 2.0, and the library is diluted to 1 ng/μL. The insert size of the library is then assessed using an Agilent 2100 Bioanalyzer. If the insert size meets the expected criteria, the effective concentration of the library is accurately quantified using qPCR (library effective concentration > 2 nM) to ensure the quality of the library. Finally, sequencing is carried out on the prepared library. When the data analysis is complete, to determine enriched pathways, gene ontology and KEGG analyses were performed using KOBAS. Furthermore, functional interaction networks are developed by examining the associations of genes targeted by PD‐L1 knockdown, utilizing protein interaction data from the STRING database. We have submitted our RNA‐seq clean data to the GEO datasets with the accession number: GSE276538. And the URL was as follows: https://www.ncbi.nlm.nih.gov/geo/query/acc.cgi?acc=GSE276538

### 2.8 Gene Enrichment Analysis

First, we selected a list of genes that showed significantly differential expression in the sequencing data. After copying the gene names or gene IDs, we opened the KOBAS website (http://bioinfo.org/kobas/genelist/). The gene list was input according to the required format. Then, the correct species for analysis was selected. Following this, KEGG or GO analysis was chosen, and the ‘RUN’ button was clicked. The enrichment analysis process was initiated. Depending on the dataset's size and the analysis's complexity, this step took some time. Upon completion of the analysis, the results provided by KOBAS were reviewed. These typically included tables and charts displaying enriched pathways, GO terms, along with associated *p*‐values or other statistical measures indicating the level of significance. Finally, the output files were saved and presented in the article.

### 2.9 RNA Isolation and RT‐qPCR

RNA was extracted from cells using Trizol (cat. no. 15596‐026; Invitrogen) according to the manufacturer's instructions. Total RNA (1 μg) was then reverse‐transcribed with the Evo M‐MLV Plus cDNA Synthesis Kit (cat. no. AG11705; Accurate Biology) reagent kit. Mixed genomic DNA (gDNA) was removed from the RNA template using gDNA Clean Reagent (included in the above‐mentioned kit) at 42°C for 2 min. The real‐time PCR was performed using High‐specificity Chemically‐colored Quantitative PCR Premix (Low ROX) (cat. no. MQ00601S; Monad Biotech Co. Ltd.) QuantStudio3 (ThermoFisher Scientific Inc.) according to the manufacturer's instructions. Gene expression was determined by using the 2^−ΔΔCt^ method. GAPDH was used as the internal control, and the primers sequence of target genes was as follows:

1. qPCR primer

SCUBE1‐Q‐F: 5′‐GAGGCTGCGACCACTTCTG‐3′

SCUBE1‐Q‐R: 5′‐GGAGCACTCGTCGATGTCC‐3′

CD53‐Q‐F: 5′‐GGCTTTGGGATCTACCTG‐3′

CD53‐Q‐R: 5′‐GGATAATCAGCAGCAGGAT‐3′

CD79A‐Q‐F: 5′‐ATCATCCTCCTGTTCTGCG‐3′

CD79A‐Q‐R: 5′‐TTTTCATCTTCATATTCATCCC‐3′

CA3‐Q‐F: 5′‐ACTGGAACCCGAAGTATAACA‐3′

CA3‐Q‐R: 5′‐GGAACTCGCCATTCTCAT‐3′

EIF3C‐Q‐F: 5′‐ACAAGGCCCATCAGCGACAG‐3′

EIF3C‐Q‐R: 5′‐GAGTGCAGAGCATGGTGGTA‐3′

GPR89B‐Q‐F: 5′‐ATGTGACTGACACGGATATT‐3′

GPR89B‐Q‐R: 5′‐TCATTCCCCAGAAACCTGAT‐3′

PRODH‐Q‐F: 5′‐GGGGAACCTCGGTGCTATGC‐3′

PRODH‐Q‐R: 5′‐AGGAGGCTCCTGGCCTTCTG‐3′

RHBDL1‐Q‐F: 5′‐GGGGTGCCCCTGGAGATGGT‐3′

RHBDL1‐Q‐R: 5′‐GCCCGCATGTCGGTGATGGA‐3′

SMIM11A‐Q‐F: 5′‐ATGAATTGGAAGGTTCTTGA‐3′

SMIM11A‐Q‐R: 5′‐TCTTTGATACATTTTCACCC‐3′

GAPDH‐F: GTGGACCTGACCTGCCGTCTAG

GAPDH‐R: GAGTGGGTGTCGCTGTTGAAGTC

For splice variant validation, we utilized RT‐PCR for analysis. We evaluated splicing events by comparing the changes in PSI (the ratio of the grayscale value of the long isoform to the total grayscale value of both the long and short isoforms). The primer sequences are as follows:

2 RT‐PCR primer

CTSB‐EX‐F: 5′‐AACCGCTCCGGCAACGCCAACC‐3′

CTSB‐EX‐R: 5′‐GCCCACGCAAGCTGCTCT‐3′

ATG13‐EX‐F: 5′‐TGGAACGACCTGTGGAAA‐3′

ATG13‐EX‐R: 5′‐AGGCTGAGGCAGGAGAAT‐3′

ATG4D‐A5SS‐F: 5′‐CATACAGCGTTTCCAGCG‐3′

ATG4D‐A5SS‐R: 5′‐AGTCCTGAGAAACGTACACCA‐3′

CCDC74B‐A3SS‐F: 5′‐CTCCGTTACAAGCTCATAAT‐3′

CCDC74B‐A3SS‐R: 5′‐AGATGGACTTGACAGACTGG‐3′

SYTL2‐ES‐F: 5′‐TTAGTGCGTAGTGCTGAA‐3′

SYTL2‐ES‐R: 5′‐TTGGAGAAATGCTGGAAC‐3′

C8orf58‐A3SS‐F: 5′‐CCAACGTACCACTTGCCATC‐3′

C8orf58‐A3SS‐R: 5′‐CGGCAGATCCGGTTGAGC‐3′

### 2.10 Primer Design (ATG4D for Example)

We first identified the chromosomal location of the exon involved in alternative splicing of ATG4D within the sequencing data. By inputting this location into the UCSC Genome Browser, we found the sequence of the skipping exon and the flanking exon. Then we determined that the accession numbers corresponding to the two splice variants are NM_032885.6 and NR_104024.2. Finally, using the Primer5 software, we designed forward and reverse primers, respectively, from the shorter exon at the 5′ end of the alternatively spliced region and the flanking exons.

### 2.11 EdU Assays

The proliferation of PC3, DU145, and LNCAP cells was detected by using Cell‐Light EdU Apollo488 In Vitro Kit (RiboBio) according to the manufacturer's protocol. Cells were photographed using a fluorescence microscope (Leica Mi8) and the number of EdU‐positive cells was counted.

### 2.12 Colony Formation Assays

For the colony formation assay, cells (1 × 10^3^ cells/well) were seeded in the 6‐cm dishes and incubated at 37°C in a humidified incubator. When cell colonies were clearly identifiable, the colonies were fixed with methanol and stained with crystal violet. After washing with water, cell colonies were photographed for statistics.

### 2.13 Chromatin Immunoprecipitation

For the chromatin immunoprecipitation (ChIP) assay to assess the binding of PD‐L1 to the SCUBE1 promoter, cells were transfected with Flag‐tagged PD‐L1 for 24 h, following which chromatin preparation and immunoprecipitation were carried out using the ChIP‐IT Express Magnetic Chromatin Immunoprecipitation Kit (ACTIVE MOTIF 53008 & 53032), according to the manufacturer's instructions. The immunoprecipitated DNA was subsequently analyzed by quantitative polymerase chain reaction (qPCR) using primers specifically targeting the human SCUBE1 promoter region. The primer sequences used in this study are as follows:

SCUBE1‐PROMOTER‐F1: GGGGTTTGCTGAACTTTCGT

SCUBE1‐PROMOTER‐R1: TTGGCTTCGGGTCTGGTC

SCUBE1‐PROMOTER‐F2: TGACGGGCCACTGTCCTTT

SCUBE1‐PROMOTER‐R2: GCCGTTTGCTGGACGTTTCT

SCUBE1‐PROMOTER‐F3: CAGCCCCAGCCTGGCCTAGA

SCUBE1‐PROMOTER‐R3: CGCTCATGGCCCGTGGC

### 2.14 Immunofluorescence Assay

Cells were seeded onto coverslips placed in six‐well plates and cultured for 24 h. After fixation with 4% paraformaldehyde (Biosharp) for 20 min, the cells were permeabilized with 0.2% Triton X‐100 for 10 min. Subsequently, the cells were washed three times with phosphate‐buffered saline (PBS), each wash lasting 5 min. To block nonspecific antibody binding, the cells were incubated with 3% bovine serum albumin (BSA) for 30 min, followed by overnight incubation at 4°C with the primary antibody against PD‐L1 (CST 13684s, 1:100 dilution) prepared in 3% BSA. The cells were then washed three times with PBS for 5 min each. Next, the samples were incubated with fluorescent‐labeled secondary antibodies at room temperature for 1 h. Nuclei were counterstained with DAPI (Abcam), and fluorescence images were captured using a Leica microscope.

### 2.15 Statistical Analysis

All data are presented as the means ± SD from at least three independent experiments. Statistical significance for each experiment was established by two‐tailed unpaired or paired *t* test, and one‐way or two‐way ANOVA, as appropriate. *p* < 0.05 was considered statistically significant. Statistical analyses were performed using Prism 9 (Graph Pad).

## 3 Results

### 3.1 High Expression of PD‐L1 in CRPC

**FIGURE 1:**
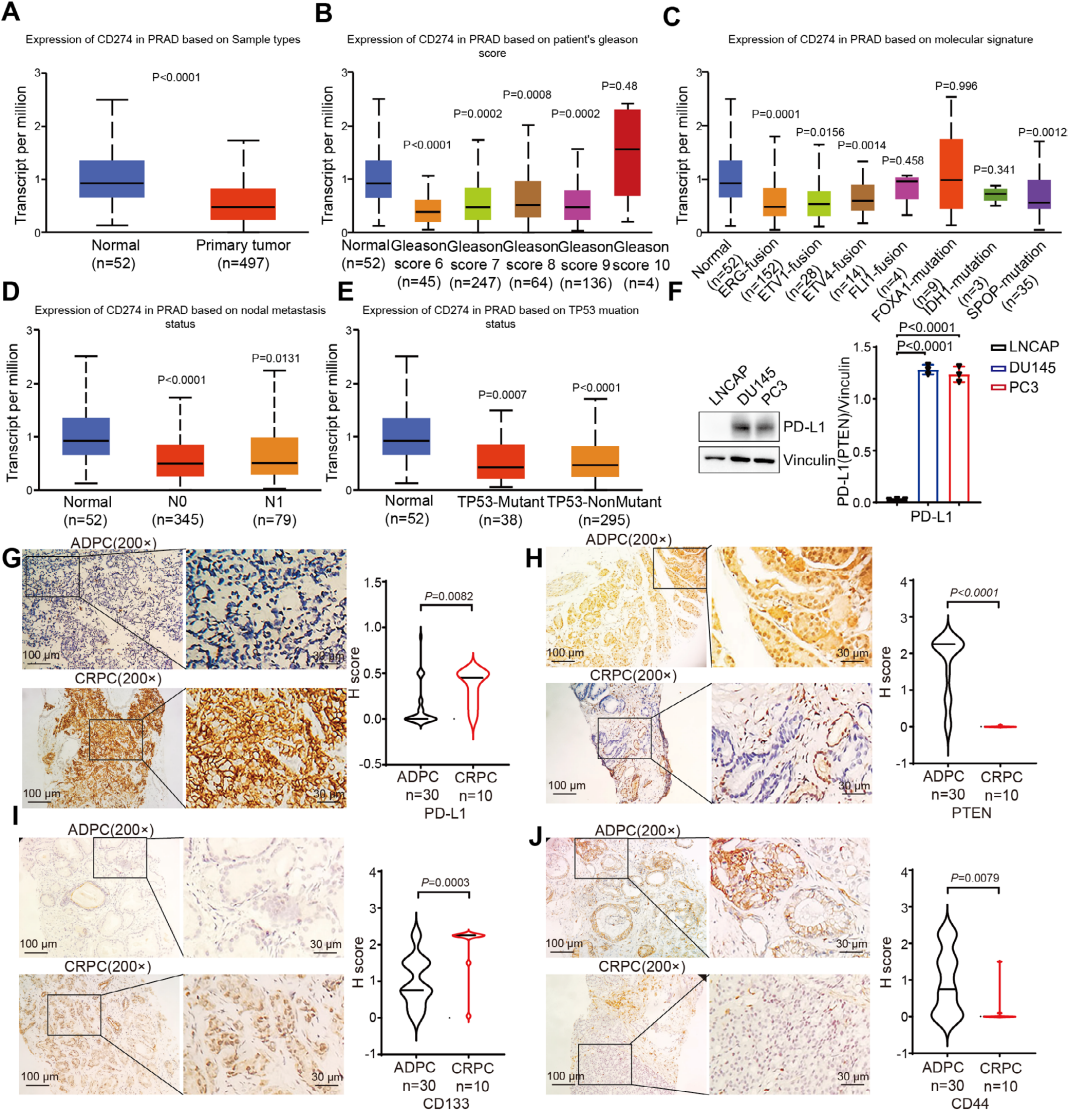
High expression of PD‐L1 in CRPC. (A–E) TCGA data analysis of CD274(PD‐L1) expression in PRAD and normal tissues based on (A) sample types, (B) patient's Gleason score, (C) molecular signature, (D) nodal metastasis status, and (E) TP53 mutation status. (F) Western blotting was used to verify PD‐L1 and vinculin protein level expression in LNCAP, DU145, and PC3 cells. The densities of signals were determined by densitometry, and three experiments were carried out with mean ± SD of change of PD‐L1/vinculin. (G–J) Representative images from immunohistochemical staining of PD‐L1 (AmoyDx cat# E1L3N) (G) PTEN (MAXIM cat# 138G6) (H) CD133 (ABCAM cat# AB19898) (I) CD44 (ZSBIO cat# OTI1D8) (J) in ADPC (*n* = 30) and CRPC (*n* = 10).

Firstly, we analyzed the expression pattern of PD‐L1 in prostate cancer from the TCGA database. Surprisingly, we found that PD‐L1 expression levels were significantly lower in prostate cancer tissues compared to adjacent normal tissues (Figure 1A), which contradicts its known function in suppressing immune responses and promoting tumor growth. This led us to hypothesize that PD‐L1 expression might correlate with clinicopathological parameters in prostate cancer. Upon further examination, we did not find any evident correlation between PD‐L1 expression levels and Gleason scores or key molecular features associated with prostate cancer (Figure 1B,C). Moreover, PD‐L1 expression was not directly related to clinical stages or P53 mutations (Figure 1D,E).

To investigate whether PD‐L1 expression is linked to castration resistance, we firstly chose the pre‐CRPC cell line LNCAP and the post‐CRPC cell lines DU145 and PC3 for investigation into the relationship between PD‐L1 expression and CRPC. Western blot analysis revealed that PD‐L1 expression was almost undetectable in the pre‐CRPC cell line (LNCAP), whereas it was notably higher in the post‐CRPC cell lines (PC3 and DU145) (Figure 1F). We proceeded to collect prostate cancer tissues from patients before and after the development of CRPC. Through immunohistochemistry, we observed that PD‐L1 was barely expressed in androgen‐dependent prostate cancer tissues (ADPC), but its expression was significantly elevated in CRPC tissues (Figure 1G). Concurrently, PTEN expression was higher in ADPC but decreased in CRPC tissues (Figure 1H). We found that CD133 and CD44 can serve as stemness markers in prostate cancer [31]. We were also interested in investigating whether there is a difference in stemness between CRPC and ADPC. To address this, we examined the expression levels of these two markers in both CRPC and ADPC. We found that the expressions of CD44 in CRPC take a downturn compared to their ADPC counterparts. However, the expression of CD133 was higher in CRPC (Figure 1I,J). Building upon these observations, we propose that PD‐L1 might play a role in the progression of prostate cancer cells post‐CRPC. The clinicopathologic feature of the ADPC and CRPC group was provided in Table 1.

### 3.2 PD‐L1 Knockdown Inhibits Proliferation of CRPC Cells

**FIGURE 2:**
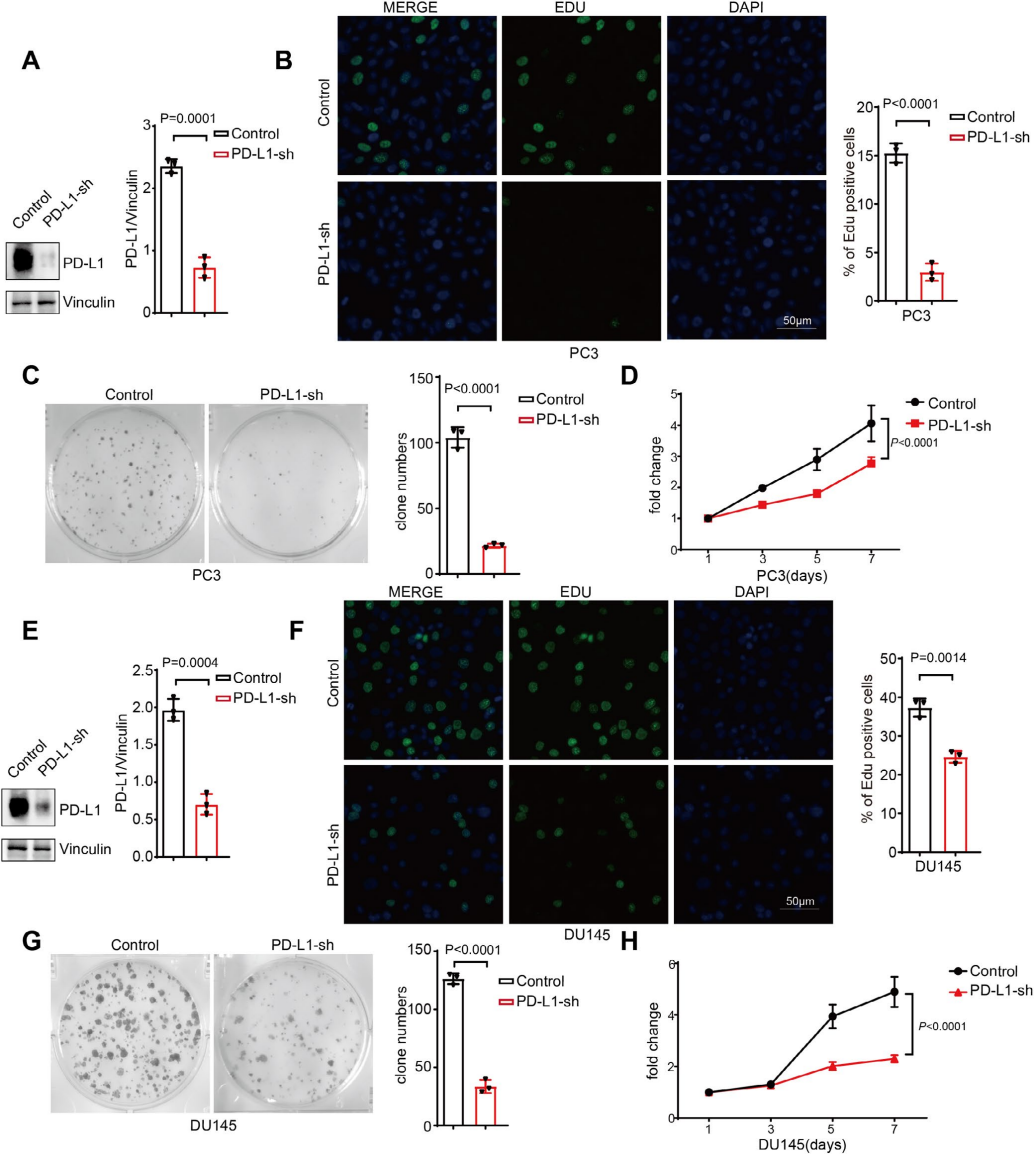
PD‐L1 knockdown inhibits proliferation of CRPC cells. (A, E) Western blotting was used to verify PD‐L1 protein level expression in PD‐L1 knockdown PC3 (A) and DU145 (E) cells. The densities of signals were determined by densitometry, and three experiments were carried out with mean ± SD of change of PD‐L1/Vinculin. (B, F) The proliferation abilities of the control, PD‐L1‐sh PC3 cells (B) and DU145 cells (F) were determined by Edu assay. (C, G) Colony formation assays were performed in the control, PD‐L1‐sh PC3 cells (C) and DU145 cells (G). Three experiments were carried out with corresponding data normalized to control and expressed as mean ± SD. *p* values were calculated by unpaired *t* test. (D, H) Growth curve assays were conducted to evaluate the effects of PD‐L1 knockdown on the proliferation of the PC3 (D) and DU145 (H) cells. *p* values were calculated by two‐way ANOVA.

To investigate the impact of PD‐L1 on the proliferation of prostate cancer cells following the development of CRPC, we first generated PD‐L1 knockdown versions of the post‐CRPC cell lines (Figure 2A–E). Employing Edu and CCK8 assays, we observed that knocking down PD‐L1 significantly inhibited the proliferation of PC3 and DU145 cells derived from CRPC cases (Figure 2B,D,F,H). Furthermore, through clonogenic assays, we confirmed that silencing PD‐L1 led to a marked reduction in the colony‐forming ability of CRPC‐derived prostate cancer cells (Figure 2C,G).

Additionally, using transwell and scratch wound healing assays (Supplementary Figure S1A–D), we demonstrated that PD‐L1 knockdown also significantly impeded the invasive and migratory capabilities of the post‐CRPC cells. These results collectively indicate that PD‐L1 plays a critical role in promoting proliferation, invasion, and migration of prostate cancer cells that have become resistant to castration and suggest that targeting PD‐L1 could be a promising therapeutic strategy in managing CRPC.

### 3.3 RNAseq Analysis of the Differential Gene Expression in CRPC Cells Following PD‐L1 Knockdown

**FIGURE 3:**
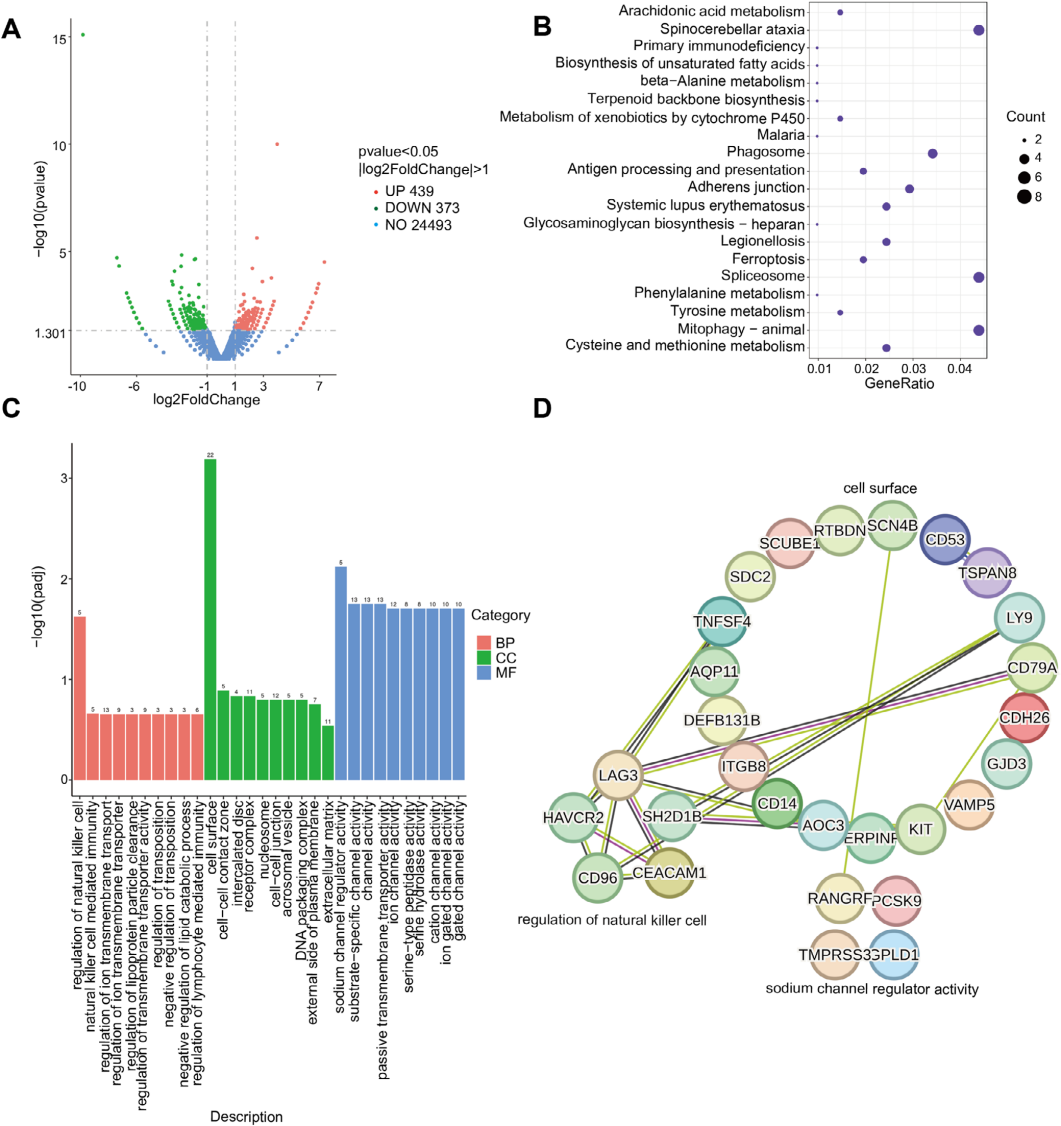
RNAseq analysis of the differential gene expression in CRPC cells following PD‐L1 knockdown. (A) Volcano plot of differentially expressed genes induced by PD‐L1 knockdown. (B) Gene ontology analyses of gene expression events. Fisher exact *p* values were plotted for each category. (C) KEGG analyses of gene expression events. Fisher exact *p* values were plotted for each category. (D) The functional association networks of differentially expressed genes induced by PD‐L1 knockdown were analyzed through the STRING database, with subgroups marked by their function.

Despite PD‐L1's typical high expression in tumors to aid in immune escape, our analysis of TCGA prostate cancer data surprisingly showed relatively low PD‐L1 expression in prostate cancer tissues, which significantly increases in castration‐resistant prostate tissues and cells. Historically, research has largely focused on PD‐L1's extracellular signaling, while its intracellular roles have been relatively understudied. Recent evidence suggests that cytoplasmic PD‐L1 can bind to mRNA to stabilize it [32], and nuclear PD‐L1 can directly interact with DNA [27]. Therefore, to explore additional roles that PD‐L1 might play in castration‐resistant prostate cancer cells beyond immune suppression, we performed RNAseq analysis using PD‐L1‐knockdown DU145 cells. The differential gene expression analysis of the sequencing data revealed 439 upregulated genes and 373 downregulated genes following PD‐L1 knockdown (Figure 3A). Function enrichment analysis of these genes disclosed that PD‐L1 knockdown led to alterations in several signaling pathways within CRPC cells, including cell surface interactions, regulation of natural killer cell activity, and sodium channel regulatory activity (Figure 3B,C). Employing the STRING database to analyze the functional relationships among the key genes involved in these pathways, we found that the main genes influenced by PD‐L1 exhibited strong correlations (Figure 3D). This indicates that PD‐L1 knockdown in CRPC cells can affect a range of cellular processes and pathways beyond its traditional immune regulatory function, shedding light on novel aspects of PD‐L1's role in CRPC progression and possibly offering new therapeutic targets.

### 3.4 Validation of Expression Levels of Key Genes Enriched in PD‐L1‐Influenced Pathways

**FIGURE 4:**
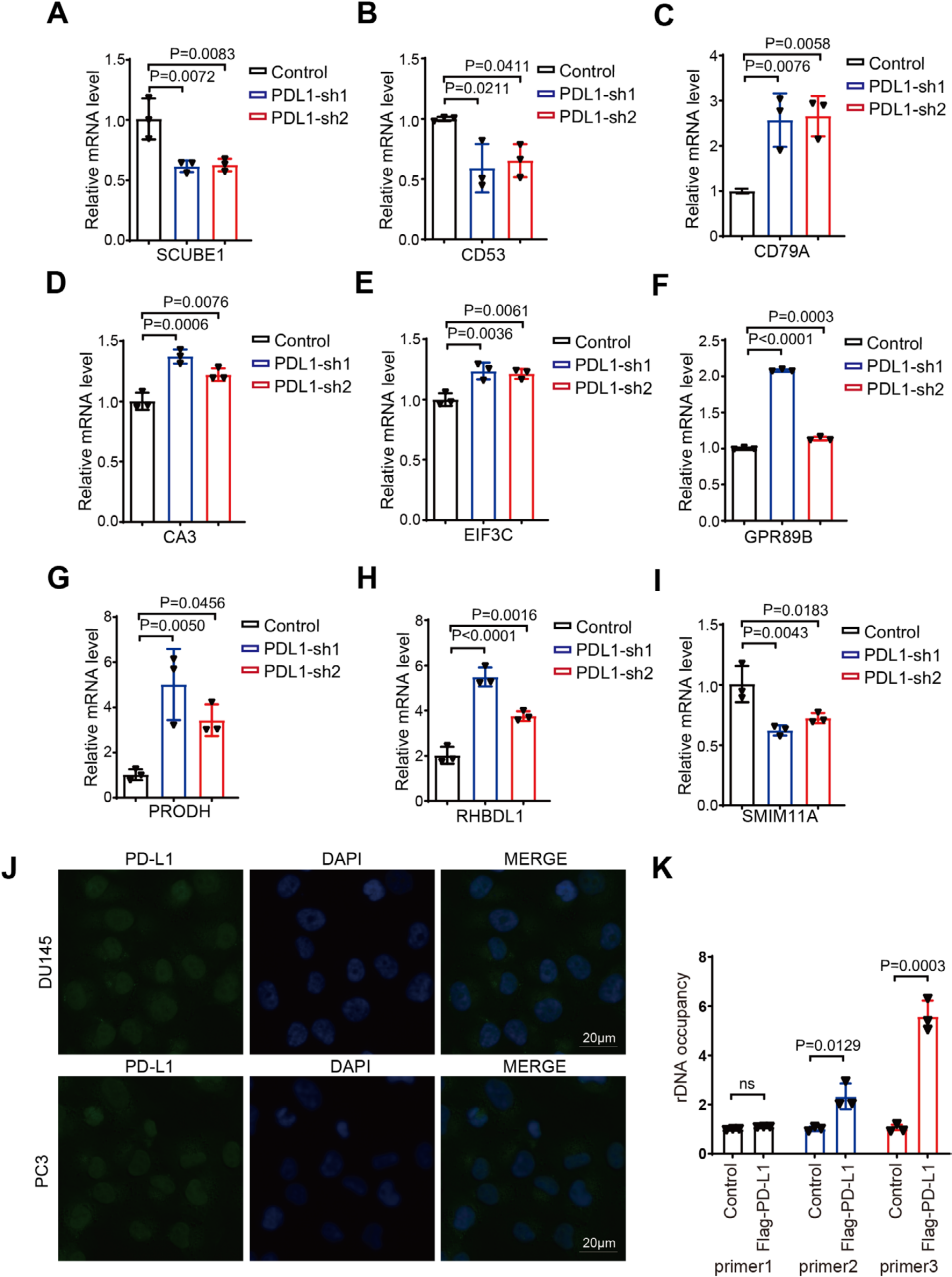
Validation of expression levels of key genes enriched in PD‐L1‐influenced pathways. (A–I) The identified genes expression changes were validated by qRT PCR. The mean SD of relative fold changes from triplicate experiments was plotted and *p* values were calculated by one‐way ANOVA. (J) The localization of PD‐L1 was examined with anti‐PD‐L1 antibodies using fluorescence microscopy in PC3/DU145 cells with stable expression of PD‐L1. (K) Binding of the SCUBE1 promoter with PD‐L1 was examined by ChIP in DU145 cells expressing FLAG‐PD‐L1.

Subsequently, to validate the RNAseq findings, we employed real‐time polymerase chain reaction (RT‐PCR) techniques. Our RT‐PCR analyses confirmed that PD‐L1 knockdown resulted in altered expression levels of several genes. The expression levels of the SCUBE1 and CD53 genes were significantly downregulated in the PD‐L1 knockdown group, whereas the expression levels of CD79A, CA3, EIF3C, GPR89B, PRODH, RHBDL1, and SMIM11A genes were significantly upregulated in the same group (Figure 4A–I). These genes were among those identified as differentially expressed in the pathways enriched following PD‐L1 knockdown in CRPC cells.

To explore the potential mechanism by which PD‐L1 influences gene expression, we first performed immunofluorescence staining to examine the intracellular localization of PD‐L1. We observed that, in addition to its presence on the cell membrane and in the cytoplasm, PD‐L1 was also partially localized in the nucleus (Figure 4J). This finding suggested that PD‐L1 might exert transcriptional regulatory functions within the nucleus. Based on this observation, we further conducted chromatin immunoprecipitation (ChIP) assays to investigate whether PD‐L1 could bind to the promoter region of SCUBE1. We divided the SCUBE1 promoter into three segments and designed specific primers for each region. Our results demonstrated that PD‐L1 exhibited strong binding affinity to the region of the SCUBE1 promoter proximal to the 5′ UTR (Figure 4K). This provides compelling evidence that PD‐L1 can function as a transcription factor involved in the regulation of SCUBE1 transcription.

### 3.5 Analysis of Alternative Splicing Events Altered by PD‐L1 Knockdown

**FIGURE 5:**
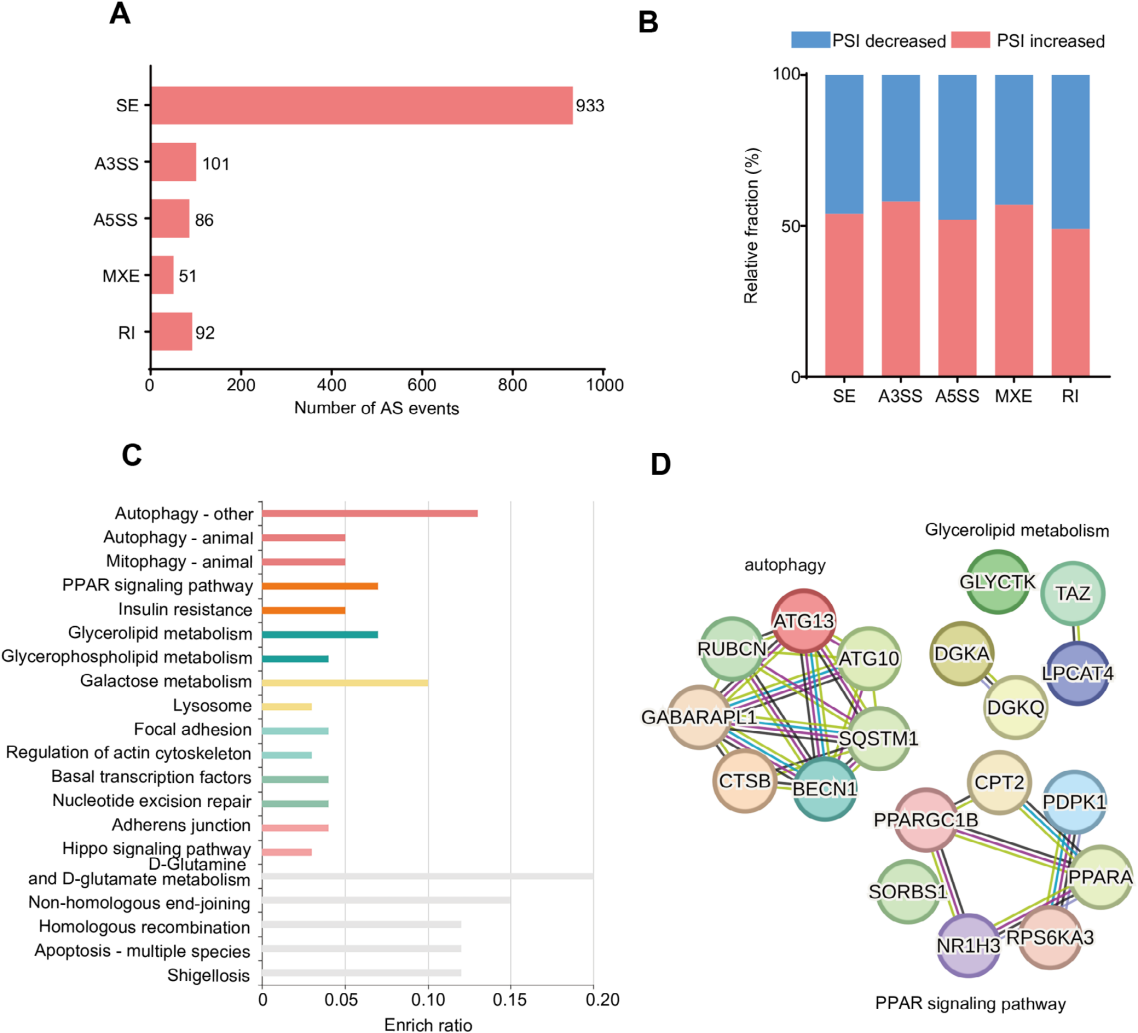
Analysis of alternative splicing events altered by PD‐L1 knockdown. (A) Quantitative statistics of alternative splicing events. (B) PSI analyses of gene alternative splicing events. (C) Gene ontology analyses of gene alternative splicing events. (D) The functional association networks of alternative splicing events induced by PD‐L1 knockdown were analyzed through the STRING database, with subgroups marked by their function.

We further delved into the potential impact of PD‐L1 knockdown on alternative splicing events. The results showed that PD‐L1 knockdown led to changes in multiple alternative splicing events, with cassette exon splicing (SE) being the predominant form, encompassing 933 differentially regulated SE events (Figure 5A). Notably, PD‐L1 knockdown did not have a clear effect on the percent spliced‐in (PSI) values, which represent the inclusion ratio of alternative exons (Figure 5B).

Gene ontology enrichment analysis of the affected splicing events indicated that they were predominantly enriched in autophagy‐related pathways (Figure 5C,D). This suggests that PD‐L1 may have a previously unrecognized role in the regulation of splicing events, particularly in the context of autophagy—a cellular process vital for cellular homeostasis and frequently deregulated in cancer. The identification of these PD‐L1‐altered splicing events opens up new avenues for understanding the complex mechanisms driving castration‐resistant prostate cancer and presents opportunities for therapeutic intervention by targeting specific splicing patterns.

### 3.6 Verification of Alternative Splicing Events Using RT‐PCR

**FIGURE 6:**
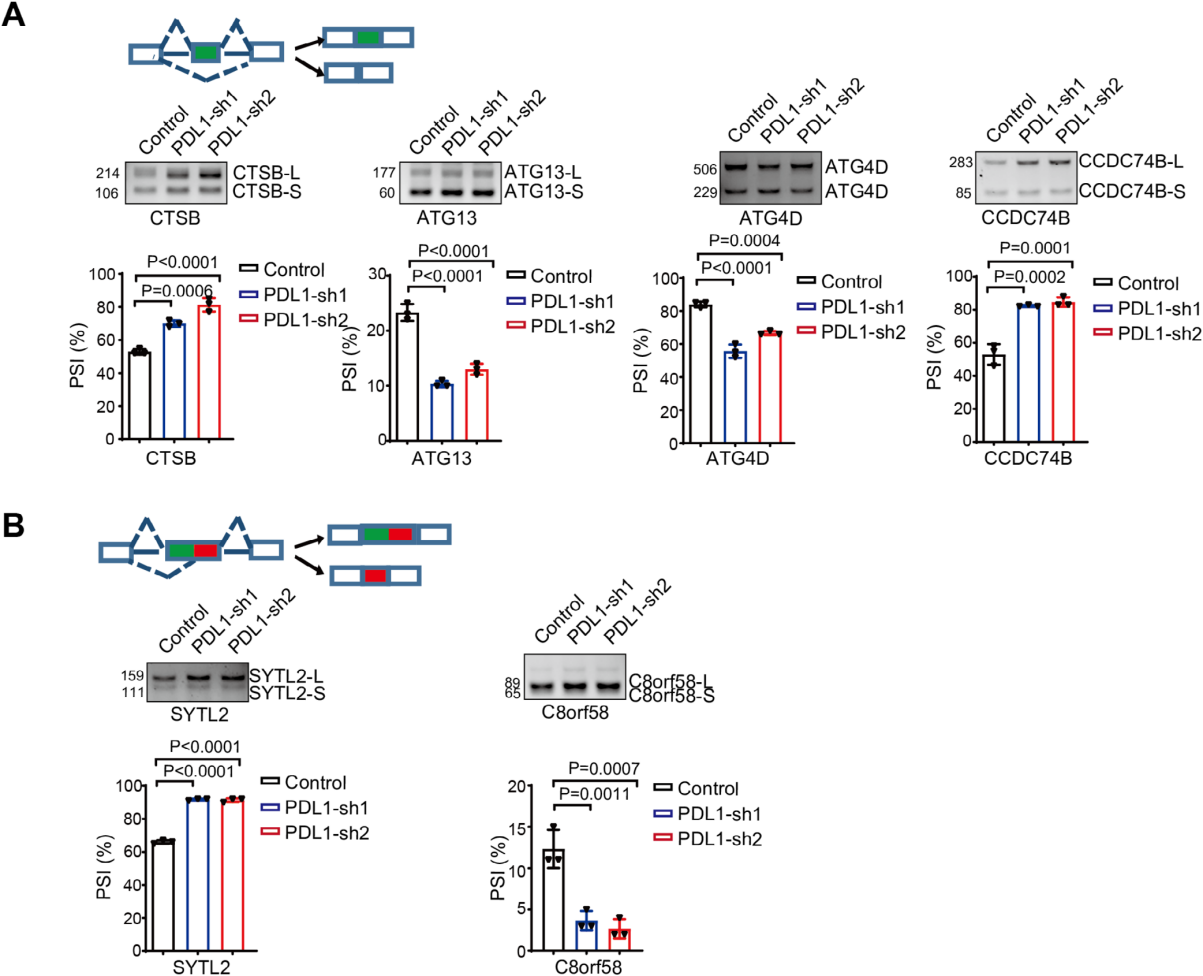
Verification of alternative splicing events using RT‐PCR. (A, B) The identified splicing events were validated by RT PCR. The mean SD of relative fold changes from triplicate experiments was plotted and *p* values were calculated by one‐way ANOVA.

To further substantiate the changes in alternative splicing events resulting from PD‐L1 knockdown, we employed RT‐PCR to verify these events. We evaluated splicing events by comparing the changes in PSI (the ratio of the grayscale value of the long isoform to the total grayscale value of both the long and short isoforms). Initially, focusing on cassette exon splicing events, we confirmed that PD‐L1 knockdown indeed affects the alternative splicing of multiple genes, including ATG13, which PSI decreased (Figure 6A). A decrease in PSI indicates a reduction in the long isoform of ATG13 and an increase in the short isoform. The long isoform of ATG13, which is the canonical splice variant, participates in the autophagy process, whereas the short isoform may lack essential regulatory functions for autophagy. This finding is indicative of PD‐L1's influence on autophagy‐related pathways, as ATG13 is a key component of the autophagy machinery. Next, we extended our validation efforts to include some alternative 3′ splice site (A3SS) events. RT‐PCR analysis verified that the knockdown of PD‐L1 resulted in altered A3SS in several genes, with SYTL2 being one such example where changes in alternative splicing were detected, which PSI increased (Figure 6B).

These validations through RT‐PCR provide empirical evidence for the role of PD‐L1 in modulating the splicing patterns of specific genes, thus adding depth to our understanding of how PD‐L1 might regulate tumor progression. By influencing the splicing of genes such as ATG13 and SYTL2, PD‐L1 may alter the functional output of these proteins, which could ultimately contribute to the development and progression of castration‐resistant prostate cancer. This information can guide future investigations into novel therapeutic strategies targeting PD‐L1‐regulated splicing events to manage CRPC more effectively.

### 3.7 PD‐L1 Contributes to the Progression of CRPC by Modulating the Expression of SCUBE1

**FIGURE 7:**
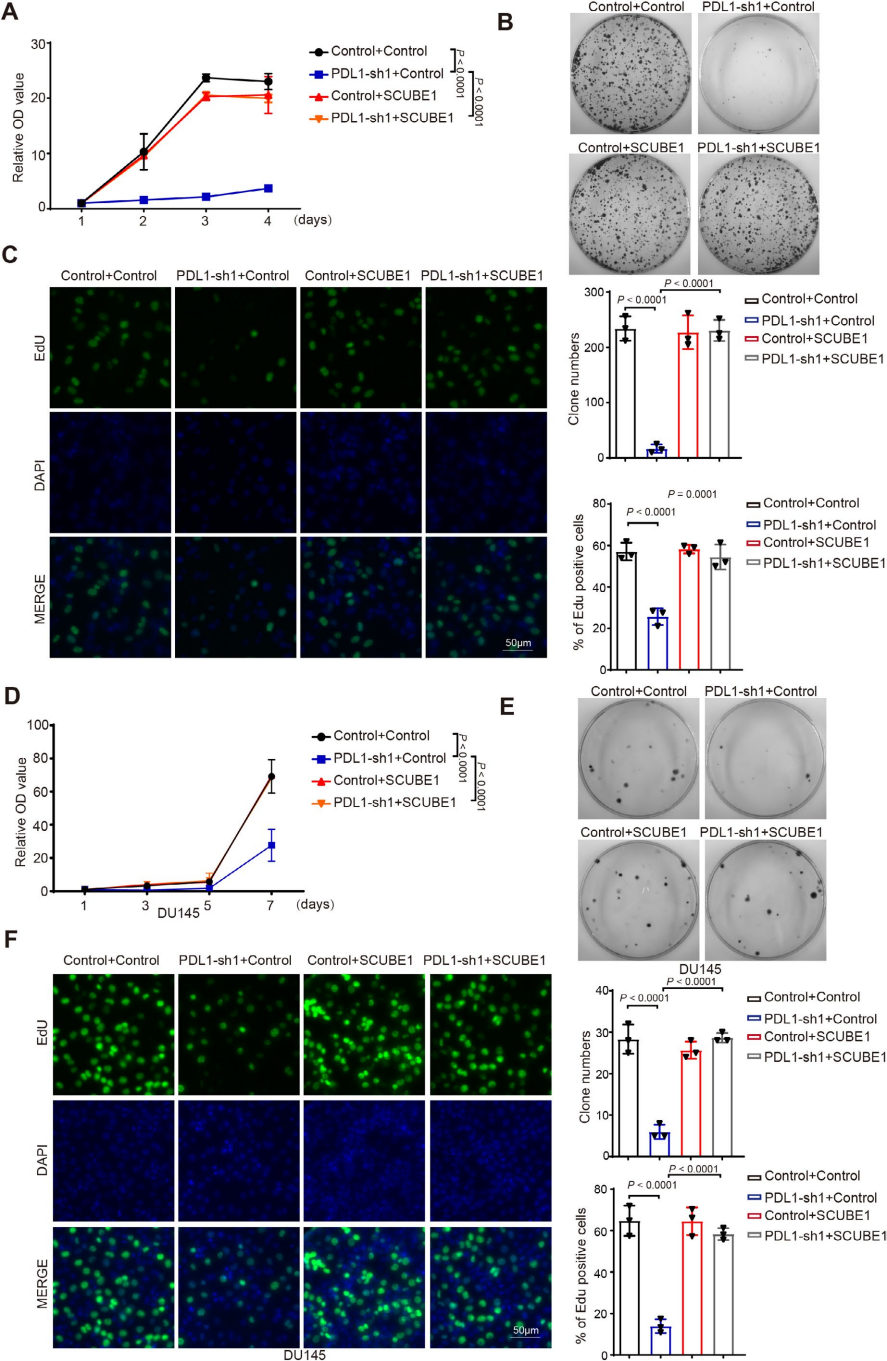
PD‐L1 contributes to the progression of CRPC by modulating the expression of SCUBE1. (A, D) CCK8 assays were performed to determine cell growth in PC3 (A)/DU145 (D) cells with stable PD‐L1 knockdown with or without SCUBE1. *p* values were determined by two‐way repeated measures ANOVA. (B, E) Colony formation assays using PC3 (B)/DU145 (E) cells with stable knockdown of PD‐L1 in the presence or absence of SCUBE1. Representative pictures of the whole plates from triplicate experiments are shown. The mean ± SD of colony numbers was plotted, with *p* values calculated by one‐way ANOVA with Dunnett's multiple comparison test, *t*‐test. (C, F) The proliferation abilities of PC3 (C)/DU145 (F) cells with stable knockdown of PD‐L1 in the presence or absence of SCUBE1. Quantification of EdU positive cells was plotted, with *p* values calculated by one‐way ANOVA with Dunnett's multiple comparison test, *t*‐test. Scale bar: 50 μm.

To investigate whether PD‐L1 influences the progression of castration‐resistant prostate cancer (CRPC) through modulating the expression of SCUBE1, we overexpressed SCUBE1 in the PD‐L1 knockdown CRPC cell line PC3/DU145. Growth curve assays revealed that overexpression of SCUBE1 significantly restored the proliferative capacity of CRPC cells inhibited by PD‐L1 knockdown (Figure 7A,D). Furthermore, colony formation assays indicated that the overexpression of SCUBE1 also significantly rescued the inhibitory effect of PD‐L1 knockdown on the clonogenic ability of CRPC cells (Figure 7B,E). Finally, Edu incorporation assays confirmed that PD‐L1 participates in the proliferation process of CRPC cells via regulation of SCUBE1 expression (Figure 7C,F).

Full‐length gels and blots were presented in Figure S2.

## 4 Discussion

Prostate cancer, as one of the most commonly diagnosed cancers worldwide, poses a persistent threat to human health. Androgen deprivation therapy (ADT) or castration treatment is often a cornerstone in the management of prostate cancer. However, the development of castration resistance often culminates in treatment failure, underscoring the importance of identifying the reasons behind this resistance. Our research has revealed that although PD‐L1 is not highly expressed in regular prostate cancer tissues, its expression levels are significantly elevated in castration‐resistant prostate cancer (CRPC) tissues and cells. This observation raises the possibility that PD‐L1 may be closely associated with the development of castration resistance.

Indeed, given the results from our experiments where we knocked down PD‐L1 in post‐CRPC cells (PC3 and DU145), we observed a marked decrease in proliferation, invasion, and migration capabilities of these cells following PD‐L1 depletion. This prompted us to speculate that PD‐L1 might have a broader role than just its established immune suppressive effects, potentially influencing intracellular pathways that drive tumor progression. To investigate the detailed mechanisms behind this phenomenon, we employed RNA sequencing (RNAseq) to perform a transcriptomic analysis on the PD‐L1 knockdown cells. The functional enrichment analysis of differentially expressed genes revealed that PD‐L1 knockdown significantly alters pathways such as cell surface interactions, regulation of natural killer cell activity, and sodium channel regulatory activity in CRPC cells. Upon further validation using PCR techniques, we confirmed that the knockdown of PD‐L1 results in changed expression levels of several genes, including but not limited to SCUBE1, CD53, and CD79A.

SCUBE1 (Signal peptide‐CUB‐EGF‐like domain‐containing protein 1) is one of the earliest identified members of the SCUBE family, characterized by high evolutionary conservation [33]. Comprised of 1000 amino acids, SCUBE1 possesses five primary protein structural domains: (1) An N‐terminal leader sequence responsible for secretion. (2) Nine tandem epidermal growth factor (EGF) like repeats, which typically mediate protein–protein interactions. (3) A large spacer region that undergoes N‐linked glycosylation, which can influence protein folding, stability, and function. (4) Three stretches of six cysteine residues (termed CR motifs) with conserved amino acid spacing between the cysteines, which are thought to be involved in disulfide bond formation, essential for maintaining protein structure and function. (5) A CUB (Complement C1r/C1s, Uegf, Bmp1) domain located at the C‐terminus, which is often associated with protein–protein interactions and modular assembly in developmental processes. While increased SCUBE1 expression has been reported in renal cancer and breast cancer, the exact role and relevance of SCUBE1 in various tumor contexts remain relatively underexplored. In leukemia research, SCUBE1 expression abnormalities have been correlated with poor prognosis. The proposed mechanism involves SCUBE1 acting as a target for HOXA9/MEIS1 and interacting with FLT3, stabilizing and facilitating activation of FLT3, thereby contributing to leukemogenesis [34, 35]. This implies that SCUBE1 might play a role in oncogenic signaling pathways, which could have implications for the progression of various cancers, including potentially in the context of castration‐resistant prostate cancer where changes in SCUBE1 expression have been observed following PD‐L1 knockdown. Our results indicated that PD‐L1 contributes to tumor progression in CRPC by modulating the expression of SCUBE1.

CD53, a member of the tetraspanin superfamily, has accumulated substantial evidence suggesting its significant role in the immune system, primarily concerning the regulation of cell adhesion. CD53 plays a part in modulating immune cell interactions; for instance, in natural killer (NK) cells, CD53 has been implicated in the induction of αLβ2 integrin activation, which leads to homotypic adhesion of NK cells [36]. This function is critical for immune surveillance and effector functions of NK cells. Moreover, CD53 has been shown to interact with various membrane proteins and participate in signal transduction pathways. For example, it is documented that CD53 can engage with CD2, thereby contributing to the activation of the phosphatidylinositol signaling pathway [37, 38]. This interaction might influence immune cell activation, proliferation, and migration, as phosphoinositide signaling is integral to numerous cellular processes, including cell motility, cytoskeletal rearrangements, and intracellular calcium release. The upregulation or downregulation of CD53 expression in various diseases, including cancer, can therefore potentially affect the tumor microenvironment and immune response. In the context of prostate cancer and specifically in castration‐resistant prostate cancer (CRPC), changes in CD53 expression following PD‐L1 knockdown may reflect alterations in the balance of immune cell interactions and signaling cascades that contribute to tumor progression or resistance to therapy. The interplay between CD53 and other molecules like PD‐L1 may provide new insights into the complex immune evasion mechanisms and therapeutic targets in advanced prostate cancer states.

CD79A is one of the two transmembrane proteins that together with CD79B form the CD79 heterodimer. Both CD79A and CD79B belong to the immunoglobulin gene superfamily [39, 40, 41]. CD79A is required in cooperation with CD79B for initiation of the signal transduction cascade activated by binding of antigen to the B‐cell antigen receptor complex (BCR) which leads to internalization of the complex, trafficking to late endosomes, and antigen presentation.

What mechanisms underlie PD‐L1's regulation of the expression levels of certain membrane proteins? Our earlier results show that PD‐L1 expression, unexpectedly, does not escalate but diminishes in prostate carcinoma tissues when compared to adjacent normal tissues. Nonetheless, a marked elevation in PD‐L1 expression is observed in both castration‐resistant cellular and tissue contexts, pointing to a probable functional role for PD‐L1 in the context of castration resistance. Thus, we utilized RNA sequencing (RNAseq) to analyze castration‐resistant prostate cancer cells, which unveiled that PD‐L1 indeed governs the alteration of expression levels across a variety of genes.

Historically, research has mainly focused on the extracellular signaling functions of PD‐L1, while its intracellular signaling roles have been relatively overlooked. Intracellular full‐length PD‐L1, despite sharing the same amino acid sequence as its extracellular counterpart, may not fully fold its N‐terminal domain, enabling it to interact with distinct proteins and biomolecules within the cell. Notably, cytoplasmic PD‐L1 has been shown to associate with mRNA and stabilize it [32]. Furthermore, in certain triple‐negative breast cancer cell lines, perinuclear or nuclear PD‐L1 interacts with DNA‐dependent protein kinase, activating MAPK or ERK pathways, thus promoting cell survival signaling independent of PD‐1 or CD80, which are conventional PD‐L1 binding partners [42]. Moreover, nuclear PD‐L1 has been suggested to directly bind to DNA [27], potentially implicating it in DNA damage repair pathways and the regulation of chemotherapy resistance. However, the precise mechanisms by which nuclear PD‐L1 influences DNA damage repair and chemoresistance require further experimental elucidation. Based on these findings, we hypothesize that PD‐L1 may modulate the expression of various genes in castration‐resistant prostate cancer (CRPC) cells through its intracellular signaling, consequently affecting the tumor phenotype of prostate cancer cells. This newly recognized aspect of PD‐L1's functionality could shed light on the mechanisms underlying the progression and treatment resistance of CRPC, opening up novel therapeutic avenues for addressing this clinically challenging disease stage.

More interestingly, we also discovered that PD‐L1 knockdown results in a multitude of changes in alternative splicing events. Gene enrichment analysis highlighted that the splicing events affected by PD‐L1 knockdown are predominantly enriched in autophagy‐related pathways. Upon verification, we indeed found that PD‐L1 can influence the alternative splicing of several genes, including ATG13 among others. These observations expand the repertoire of potential intracellular signaling mechanisms orchestrated by PD‐L1, demonstrating that its impact extends beyond classical immune checkpoint pathways to include modulation of RNA processing and autophagic processes. This revelation offers new insights into the multifaceted role of PD‐L1 in cancer progression, particularly in the context of castration‐resistant prostate cancer, and emphasizes the importance of considering its broader regulatory functions within the cell.

This study pioneers the use of RNAseq technology to investigate the impact of PD‐L1 on the resistance and proliferation of prostate cancer cells following the development of castration resistance (CRPC). Our findings reveal that PD‐L1 has the capacity to influence the expression of multiple genes and may participate in the regulation of alternative splicing events. These discoveries lay a foundation for further exploration into the multifaceted roles of PD‐L1 in tumor biology and provide rationale for the development of PD‐L1‐targeted therapeutics in clinical settings. This work underscores the significance of PD‐L1 beyond its known immune checkpoint function and highlights its potential involvement in molecular pathways contributing to treatment resistance and tumor progression in CRPC.

## Author Contributions

**Lin Zhong:** investigation; methodology; writing – original draft; writing – review and editing. **Pengxin Zhang:** investigation; software. **Jialin Ji:** investigation; writing – review and editing. **Jun Mao:** investigation. **Lianhong Li:** funding acquisition; methodology; investigation; writing – original draft; writing – review and editing.

## Ethics Statement

The present study was approved by the Institutional Review Board of the First Affiliated Hospital of Dalian Medical University in accordance with the Declaration of Helsinki.

## Consent

Written informed consent was obtained from each participant. All experimental protocols are in accordance with the International Convention on the Ethics of Laboratory Animals and relevant national regulations.

## Conflicts of Interest

The authors declare no conflicts of interest.

## Supporting information


**Figure S1:** PD‐L1 knockdown inhibits metastatic of CRPC cells.
**Figure S2:** Full‐length gels and blots.

## Data Availability

The data that support the findings of this study are available on request from the corresponding author. The data are not publicly available due to privacy or ethical restrictions.
