# Supplementary material for: Exploring the Functional Role of Programmed Death‐Ligand 1 (PD‐L1) in the Castration‐Resistant Prostate Cancer Using Transcriptomic Sequencing Analysis
Source: Cancer Med. 2025 Sep 9;14(17):e71225. doi: 10.1002/cam4.71225 (PMC12418085; doi:10.1002/cam4.71225)
Supplement: Supplementary file 1 — Figure S1: PD‐L1 knockdown inhibits metastatic of CRPC cells. Figure S2: Full‐length gels and blots. [file CAM4-14-e71225-s001.docx]

**
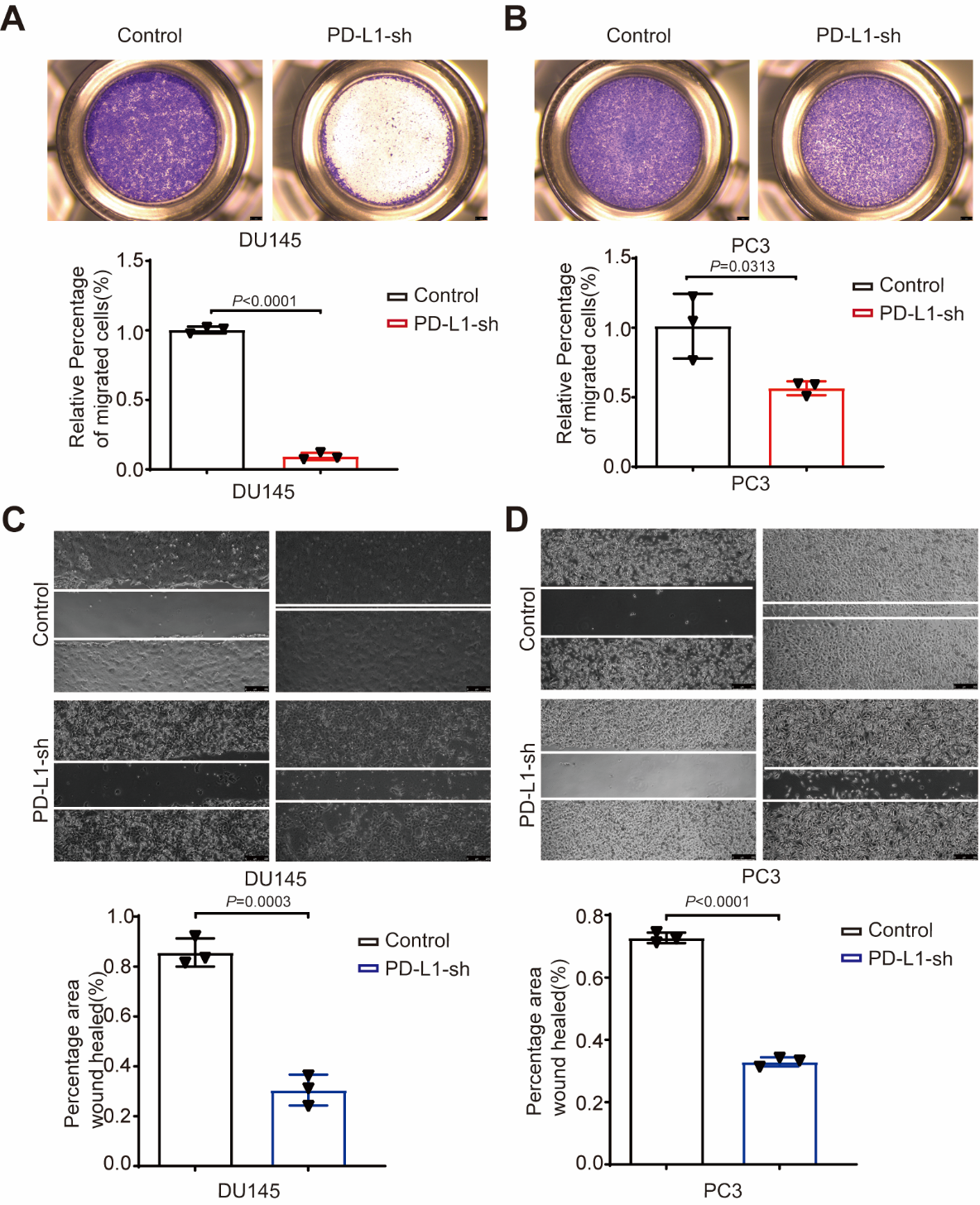
**

Supplementary Figure1：PD-L1 Knockdown Inhibits metastatic of CRPC Cells

(A B) Transwell assays were performed in the control, PD-L1-sh DU145 cells (A) and PC3 cells (B). (C D) Wound Healing were performed in the control, PD-L1-sh DU145 cells (C) and PC3 cells (D).


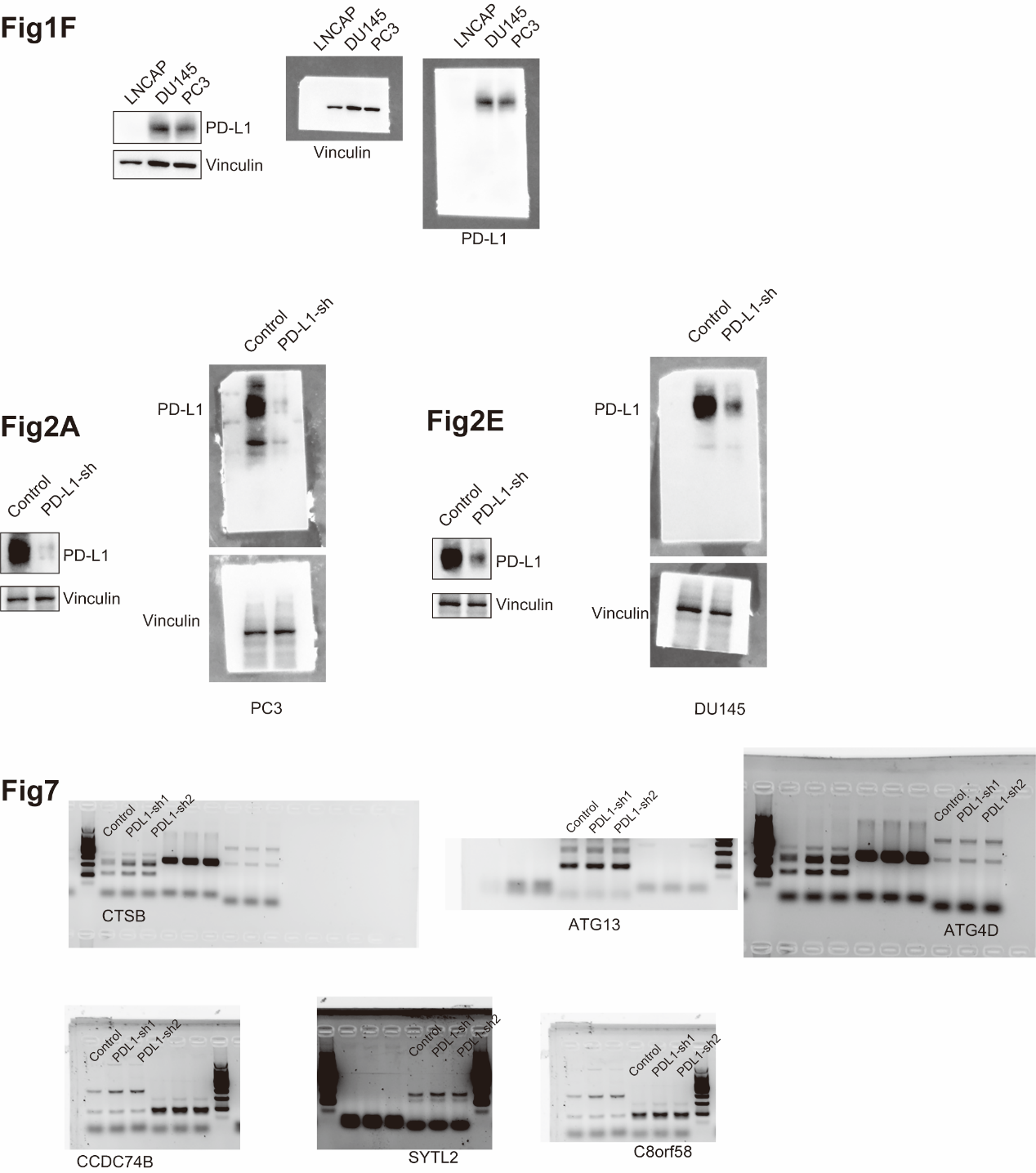


Supplementary Figure2: Full-length gels and blots.
